# Electron-Beam-Induced Modification of N-Heterocyclic Carbenes: Carbon Nanomembrane Formation

**DOI:** 10.1021/acs.jpclett.4c01705

**Published:** 2024-08-02

**Authors:** Daria M. Cegiełka, Martha Frey, Krzysztof Kozieł, Christof Neumann, Andrey Turchanin, Piotr Cyganik

**Affiliations:** † Jagiellonian University, Faculty of Physics, Astronomy and Applied Computer Science, Smoluchowski Institute of Physics, Łojasiewicza 11, 30-348 Krakow, Poland; ‡ Jagiellonian University, Doctoral School of Exact and Natural Sciences, Łojasiewicza 11, 30-348 Krakow, Poland; § Institute of Physical Chemistry, Friedrich Schiller University Jena, Lessingstraße 10, 07743 Jena, Germany; ∥ Faculty of Chemistry, Jagiellonian University, 30-387 Krakow, Poland; ⊥ Jena Center for Soft Matter, 07743 Jena, Germany

## Abstract

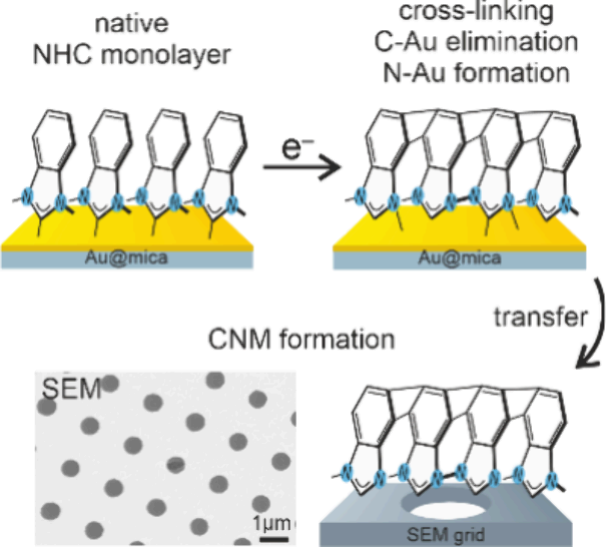

Electron irradiation of self-assembled monolayers (SAMs) is a versatile tool for lithographic methods and the formation of new 2D materials such as carbon nanomembranes (CNMs). While the interaction between the electron beam and standard thiolate SAMs has been well studied, the effect of electron irradiation for chemically and thermally ultrastable N-heterocyclic carbenes (NHCs) remains unknown. Here we analyze electron irradiation of NHC SAMs featuring different numbers of benzene moieties and different sizes of the nitrogen side groups to modify their structure. Our results provide design rules to optimize NHC SAMs for effective electron-beam modification that includes the formation of sulfur-free CNMs, which are more suitable for ultrafiltration applications. Considering that NHC monolayers exhibit up to 100 times higher stability of their bonding with the metal substrate toward electron-irradiation compared to standard SAMs, they offer a new alternative for chemical lithography where structural modification of SAMs should be limited to the functional group.

Self-assembled monolayers (SAMs) form well-defined organic nanostructures on inorganic substrates by spontaneous chemisorption.^1−3^ The ease of SAM formation provides a simple and extremely effective method for surface/interface functionalization, which was recently recognized by awarding the Kavli Award in Nanoscience to the discoverers of SAMs.^4^ This functionalization process becomes particularly robust in combination with several pattering methods of SAMs^5^ which were applied mainly to build organic electronic devices^6−14^ and control biocompatible interfaces toward the design of new sensors and tissue engineering.^15−18^ In particular, electron-beam irradiation allows for well-defined modification of the structure of the SAM, and thus related surface/interface functionalization via several different processes that include partial desorption of monolayer,^19−21^ cross-linking of molecules within the monolayer,^22−26^ and modification of their chemical functionality.^27−31^ These electron-beam stimulated processes can be operated from micrometer down to nanometer scale and were already applied for a few different types of lithography,^27,30,32−37^ work function adjustment,^38^ and formation of carbon-based materials such as graphene^39,40^ or carbon nanomembranes (CNMs).^41−45^ Generally, over the last 25 years most applications related to electron irradiation of SAMs were based on using mainly aromatic or aliphatic thiols on a gold substrate. Only recently, another approach was taken by us^46,47^ and others^48,49^ using SAMs with carboxylic anchoring group formed on a silver substrate. It was demonstrated that this type of SAMs can be applied for the formation of sulfur-free CNMs^46^ which can be favorable for some ultrafiltration processes.^50,51^ A disadvantage of this system is, however, a much lower stability of the carboxylic anchoring group toward electron irradiation compared to standard thiols,^47^ which may impair certain applications, especially those where irradiation should lead only to chemical modification of SAMs functionality and/or structure without pronounced film desorption.^29,36,38,52,53^

Searching for monolayers, which are sulfur-free but at the same time exhibit high stability of their anchoring group toward electron irradiation, one could consider N-heterocyclic carbenes (NHC) as a potential candidate. The NHC-based SAMs on gold, with the carbene carbon atom as the anchoring group, were widely explored in recent years, revealing extremely high chemical,^54^ electrochemical,^54−56^ and thermal^55,57−59^ stability compared to standard thiols on the same substrate. All these stability-related parameters are obviously crucial for most possible applications, and therefore, intensive research of NHC SAMs structure, properties, and possible applications was undertaken as documented in the literature.^60−63^ To the best of our knowledge, until now, however, the interaction of NHC SAMs with electron irradiation has not been analyzed. As argued above, optimization of electron-induced modification of a given type of SAMs opens up a wide range of their possible applications. Considering structural modifications introduced by the NHC SAMs compared to standard monolayers based on thiols or carboxylic acids, *i.e*., the new type of surface anchoring group in combination with the N-heterocyclic structure of the molecular backbone, such an analysis for NHC SAMs seems also to be crucial from the viewpoint of fundamental mechanisms of electron interaction with organic films.

To this end, in the current study, we analyze low-energy (50 eV) electron irradiation for a series of well-ordered, densely packed model NHC SAMs on gold with a systematically modified molecular structure (Figure 1). Our experiments indicate not only a much higher stability of the carbene anchoring group toward electron irradiation as compared to thiols but also electron-beam-stimulated formation of new chemical bonds with the substrate. Moreover, we also demonstrate that, for properly designed NHC SAMs, successful sulfur-free CNM formation can be achieved.

**Figure 1:**
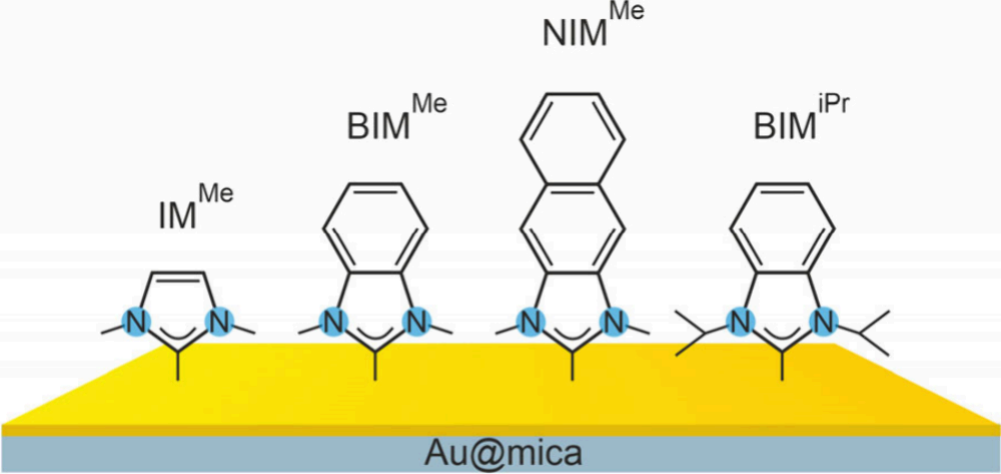
Schematic presentation of NHC molecules used for this study together with respective acronyms.

The electron irradiation experiments were performed for a series of NHC monolayers containing the imidazolium (IM) moiety to link molecules with the gold substrate, which, with only a few exceptions,^64−66^ is currently the most common strategy for NHC SAM formation.^56,60−63,67−70^ In the analyzed series of molecules the IM moiety was linked with different numbers (*n*) of benzene rings: IM^Me^ (*n* = 0), BIM^Me^/BIM^iPr^ (*n* = 1), and NIM^Me^ (*n* = 2). Importantly, in most of the systems analyzed here (IM^Me^, BIM^Me^, and NIM^Me^) short methyl (Me) side groups of nitrogen heteroatoms were used. Recent structural analysis of these monolayers^59^ demonstrated that, in contrast to the conclusions reached by former studies,^57,71−76^ NHC molecules with short Me side groups can form densely packed, upright oriented monolayers on the Au(111) substrate. To determine the possible impact of NHC with more bulky side groups, we also analyzed BIM^iPr^, which is the analogue of BIM^Me^ with isopropyl (iPr) side groups forming monolayers with roughly twice lower packing density.^59,72^

The electron irradiation of the NHC SAMs formed by the molecules presented in Figure 1 was monitored *in situ* using XPS analysis conducted for a progressively increased irradiation dose (see the Supporting Information for technical details). The series of C 1s spectra obtained for the BIM^Me^/Au monolayer is presented in Figure 2a as a representative example for all NHC SAMs analyzed here. The nonirradiated BIM^Me^/Au SAM is characterized by an asymmetric C 1s peak with the maximum of the envelope located at the binding energy (BE) of ∼285.2 eV. As discussed in our previous study^58,59^ this asymmetry is related to the presence of two main components related to the imidazolium moiety (∼286 eV) and the phenyl ring (∼285 eV). We note that the BE associated with the phenyl component is shifted by ∼0.9 eV toward higher energy, compared to data known for aromatic thiol or selenol-based SAMs (∼284.1 eV).^77−80^ Following the former analysis^58,59^ we attribute the shift of this component to an electrostatic effect^81^ caused by the formation of the dipole layer at the molecule–metal interface upon modification of the anchoring group from thiols to carbene. The observed shift toward higher BE values would then require an upward orientation of such a dipole layer (with respect to the Au substrate), which is consistent with the theoretically expected charge rearrangement upon C–Au bond formation (positive charge located at the carbene carbon atom)^82,83^ and former secondary ion mass spectrometry (SIMS) analysis.^58,59^

**Figure 2:**
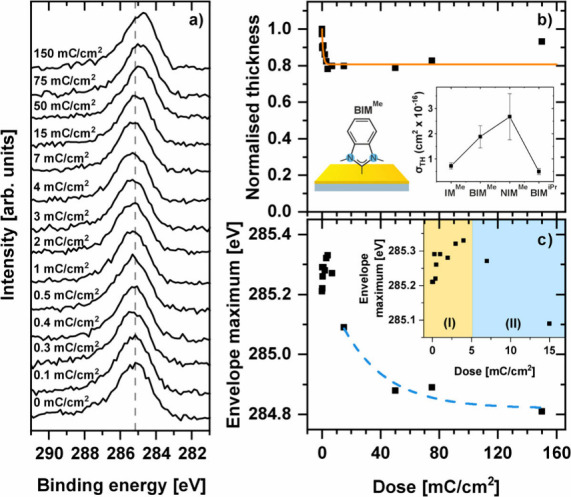
(a) C 1s XPS data for electron-beam (50 eV) irradiated BIM^Me^/Au monolayers using electron doses in the range 0–150 mC/cm^2^. The gray dashed line indicates peak position for nonirradiated (native) sample. (b) Normalized film thickness of BIM^Me^/Au as a function of the electron dose with indicated (orange line) fitting of the saturation function (eq 1). The inset shows calculated cross sections for thickness modification (σ_TH_) obtained for all analyzed NHC SAMs. (c) BE of C 1s peak (envelope) maximum as a function of the total electron dose for BIM^Me^/Au. The inset shows close-up for low doses indicating increase (by the cross-linking process, yellow range) and decrease (by the Au–C bond termination, blue range) of the BE value. The blue dashed line in panel c shows fitting of the saturation function (an analogue of eq 1) to the data in the range of 15–150 mC/cm^2^ where the Au–C bond termination process takes place.

As shown in Figure 2a, the progressive irradiation of NHC SAMs, up to a dose of 150 mC/cm^2^, leads to modification of both the intensity and position of the C 1s peak. The systematic analysis of the film thickness (calculated by a standard approach based on the C 1s/Au 4f intensity ratio)^21,47,59^ as a function of the irradiation dose *Q*/*S* [C/m^2^] is presented in Figure 2b (see Figure S1 for all NHC SAMs) and reveals the reduction of this parameter which, when normalized to the native film thickness *d*_0_, can be described by the following saturation function:^20,21,24,47,84^
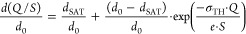
1where *d*_SAT_ and σ_TH_ are the thicknesses for the extensively irradiated monolayer (saturation value) and the cross section for this process, respectively. The fractions of thickness reduction (*d*_0_ – *d*_SAT_)/*d*_0_ and σ_TH_ parameters are summarized in Table 1. We observe ∼20% thickness reduction for all NHC SAMs except NIM^Me^/Au for which a value of ∼10% was estimated. Generally, the process of electron irradiation induced thickness reduction of SAMs is a consequence of competition of two main processes, i.e., (i) scission of existing chemical bonds within molecules (e.g., C–H and C–C), followed by the desorption of respective fragments, and (ii) formation of new C–C/C=C bonds between neighboring molecules and thus cross-linking monolayer structure, which is stabilizing it toward further desorption. For electron irradiation (50 eV) of aliphatic thiols on gold, a substantial reduction of SAMs thickness by ∼40% was reported,^20,21^ indicating the dominant role of the desorption process.

**Table 1:** Overview of the Parameters for the Analyzed NHC SAMs

Parameter		IM^Me^	BIM^Me^	NIM^Me^	BIM^iPr^
σ_TH_ (cm^2^ ×10^–16^)		0.7(0.1)	1.9(0.4)	2.7(0.9)	0.5(0.1)
σ_C-Au_ (cm^2^ ×10^–18^)	from C 1s	2.9(0.6)	5.4(3.2)	1.6(0.2)	4.0(1.4)
	from N 1s	5.0(0.8)	6.2(1.2)	1.4(0.2)	2.5(1.6)
Thickness reduction (%)		20(7)	19(8)	10(7)	24(7)
ΔBE (eV)		0.70(0.1)	0.52(0.1)	0.54(0.1)	0.77(0.1)

In contrast, for purely aromatic thiols on gold, the reported reduction of film thickness (under the same electron irradiation conditions) is much lower, and depending on the specific system, thickness reductions between nearly zero level (within the precision of such measurements ∼5%) and ∼25% were reported,^84,85^ confirming the dominant role of the cross-linking process in this case. Therefore, concerning ∼10–20% thickness reduction for the NHC monolayers here analyzed, we conclude the dominant contribution of the cross-linking process for this type of SAMs as well. Importantly, we note that even for the IM^Me^/Au monolayer, which does not contain a phenyl ring, the N-heterocyclic moiety alone provides efficient cross-linking of this monolayer with a reduction of film thickness ∼20%. This value is even lower compared to the value (∼25%) reported^85^ under the same irradiation conditions for biphenyl thiols on gold—the archetypical SAMs for electron-based lithography^27,32,34^ and carbon-based materials formation.^39,41−45^

The lowest reduction of the film thickness (∼10%) was observed for NIM^Me^/Au, which is most probably related to the longest molecular backbone and thus the highest probability for the cross-linking during the transition of electrons (primary and secondary) through the thickest monolayer. This assumption is also fully consistent with the highest value of the cross section for the thickness modification for NIM^Me^/Au among all NHC monolayers analyzed here (see the inset in Figure 2b and the data in Table 1). The higher the value of σ_TH_, the lower the dose of electron irradiation needed to reach the saturation range of film thickness reduction associated with the completeness of the monolayer cross-linking process. Therefore, σ_TH_ can be considered as a measure of the cross-linking efficiency. The systematic increase in the value of σ_TH_ with elongations of the NHC molecules skeleton going from IM^Me^/Au (∼0.7 × 10^–16^ [cm^2^]) to BIM^Me^/Au (∼1.9 × 10^–16^ [cm^2^]) and NIM^Me^/Au (σ_TH_ ≈ 2.7 × 10^–16^ [cm^2^]) confirms the above argumentation (see inset in Figure 2b). This trend does not hold for the BIM^iPr^/Au monolayer showing a value of σ_TH_ (∼0.5 × 10^–16^ [cm^2^]) which is not only much lower compared to the equally long analogues BIM^Me^/Au but also lower than the value obtained for the shortest monolayer, i.e., IM^Me^/Au. However, this is only an apparent inconsistency; in fact, this result confirms the above model considering the much more bulky nitrogen side group of the molecules forming BIM^iPr^/Au compared to all other NHC SAMs analyzed here. As pointed out earlier, the modification of this side group from Me to iPr leads to almost twice lower packing density,^59,72^ thus hindering the cross-linking process taking place between neighboring molecules in the monolayer. This observation is particularly important considering that the packing density of NHC SAMs also depends on their preparation method, i.e., in solution, in vacuum, or electrochemical.^59,63,70,86^

Another important parameter which traces the C 1s signal modification and thus the electron-beam-induced processes in the NHC films is the respective BE, which as a function of the irradiation dose is presented in Figure 2c and Figure S2. As exemplified by the data obtained for BIM^Me^/Au, the modification of the BE for the C 1s signal does not show the monotonic behavior reported earlier for SAM with thiol^20,84^ or carboxylic^47^ anchoring groups. The initial irradiation up to the dose of ∼2–4 mC/cm^2^ leads to an increase in the BE in the range of ∼0.1–0.2 eV (see the inset in Figure 2c and the data shown in Figure S2). A similar increase of the C 1s BE induced by electron irradiation (50 eV) was reported in previous experiments for purely aromatic thiols on gold with a saturation point at ∼10 mC/cm^2^.^84^ Following these former studies, we attribute the observed increase in the C 1s BE, which is not observed for purely aliphatic SAMs,^20^ to the cross-linking process taking place in the NHC SAMs. We also note that in Figure 2c the range of total electron dose where an increase in the C 1s BE was observed is the same as the range where the drop of the film thickness is taking place, which additionally supports the above hypothesis, considering that thickness modification is limited by the cross-linking process. However, in contrast to electron irradiation of all other SAMs, for NHC monolayers this initial, and relatively small, increase of C 1s BE for doses up to 3–4 mC/cm^2^ is followed by a few times larger decrease of BE, which saturates at the doses which exceed ∼100 mC/cm^2^. This observation indicates yet another process of NHC SAM modification that plays a dominant role for larger irradiation doses. Considering the direction of the BE shift toward lower values, such modification is most probably related to the elimination of the original C–Au bonds in the monolayer, which leads to destruction of the dipole layer formed upon chemisorption and as a result, progressive shifting down of the C 1s BE toward a value which is typical for cross-linked aromatic thiol systems, i.e., ∼284.2 eV.^84^ The C–Au bond termination as a function of the electron dose can be described by the respective saturation function, similar to the thickness modification discussed above. To extract the corresponding cross section for this process (σ_C-Au_) we have fitted respective data in the range from 15 to 150 mC/cm^2^, i.e., in the region of electron dose where the cross-linking process is at the saturation stage, and further modification of the BE value is due to the Au–C bond termination process only (see blue dashed line in Figure 2c and Figure S2). The estimated values of σ_C-Au_ obtained for all NHC SAMs analyzed here are in the range of ∼10^–18^ cm^2^, which is approximately 1 or 2 orders of magnitude lower than the corresponding cross-sectional values for the anchoring group termination reported previously for thiols on gold substrate^21,84^ and carboxylic acids on silver,^47^ indicating thus significantly higher stability of the C–Au bond toward electron irradiation compared to S–Au or O–Ag.

The shifting of the C 1s BE value toward lower values is directly related to the change in the potential energy step Δ*V* associated with the dipole layer formation, which from simple electrostatic considerations (omitting depolarization effects) is proportional to the surface density ρ of the dipole moments μ_CAu_ associated with the C–Au bond formation according to the following equation:^87−89^
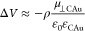
2where μ_⊥CAu_ is the normal component (to the metal substrate) of the μ_CAu_, ε_0_ is the vacuum permittivity, and ε_CAu_ is the dielectric constant at the C/Au interface. Therefore, the observed reduction in the BE (ΔBE) from the maximal value of the BE (BE_MAX_) down to the minimal value (BE_MIN_), both measured in Figure 2c, is proportional to the fraction of the C–Au bond eliminated during irradiation. The respective data summarized in Table 1 show lower values of the ΔBE parameter for BIM^Me^/Au and NIM^Me^/Au compared to the IM^Me^/Au and BIM^iPr^/Au monolayers, i.e., lower fraction of C–Au bonds terminated for the former monolayers. The higher stability of the C–Au bond toward electron irradiation for BIM^Me^/Au and NIM^Me^/Au compared to IM^Me^/Au and BIM^iPr^/Au concluded here could be correlated with previous analysis^59^ of these NHC SAMs showing much higher thermal stability for the former group of monolayers. The pronounced difference in thermal stability originates most likely from the changes in the exact configuration of the chemical bonds at the molecule–metal interface including the C–Au bond, *e.g*. different orientation of this bond relative to the metal substrate, and/or different configuration of the bonding to Au atom/adatom. The ionization and excitation processes induced by electron irradiation may lead to the formation of the repulsive state, and subsequently to dissociation of the given chemical bond.^90,91^ For chemical bonds located at the molecule–metal interface, the probability of this dissociation process can be radically reduced by nonradiative relaxation of the respective repulsive state via two processes: (i) interaction of the oscillating dipole (representing excited repulsive state) and its image, which leads to the creation of phonon or electron–hole pair in the metal substrate and (ii) electron tunneling from this excited state to the metal substrate.^91^ Since the efficiency of both processes strongly depends on the distance between the excited bond and the metal surface, as well as on the details of their electronic structure,^91^ the modification of the bonding geometry of the NHC molecules will have pronounced impact on the stability of their C–Au bond toward electron irradiation, as it was observed also for other types of SAMs.^24,47,90^

Electron-induced modification of the NHC SAMs is also visible in the N 1s signal. In Figure 3a, a representative example of such data for BIM^Me^/Au is presented. For nonirradiated (native) NHC SAMs, a single N 1s component was observed at ∼400.4 eV except for IM^Me^/Au where a slightly higher BE (∼400.7 eV) value was measured in accordance with the former analysis of these monolayers.^59^ We note at this point that due to the much lower abundance of nitrogen in NHC SAMs compared to carbon and the higher attenuation of the emission for respective photoelectrons, the signal-to-noise ratio for N 1s is significantly lower compared to C 1s, so the respective analysis is much less precise. However, the key aspects of electron beam-induced modification of NHC SAMs can be observed in the N 1s signal. As presented in Figure 3b and Figure S3, whereas the total N 1s intensity remains essentially unchanged during the irradiation process (within the ∼10% precision of our analysis), the intensity of the main component is reduced by ∼20–25% upon reaching high electron doses (50–300 mC/cm^2^) with, however, the simultaneous appearance of a new component (red dashed line in Figure 3a). This new component is located at ∼398–399 eV and, considering former XPS data for SAMs on Au(111) with the amine^92,93^ or pyridine^94^ anchoring group, can be associated with the N–Au bond formation. Although we cannot exclude that complete detachment of nitrogen from the imidazolium unit takes place upon the formation of this bond, such a scenario seems much less probable as the main component in this case would be expected at lower BE (∼397 eV), which is characteristic for gold nitride formation.^95^ The main N 1s component exhibits also the change in the BE position during irradiation which has a similar character as for the C 1s signal discussed above; that is, for low irradiation doses up to ∼5 mC/cm^2^ an increase (∼0.1–0.2 eV) is observed which for higher doses is followed by a decrease showing saturation behavior (Figure 3c). The initial increase of the N 1s BE position is most probably caused by the cross-linking mechanism and has been previously reported for electron irradiation (50 eV) of pyridine and pyrimidine substituted aromatic thiols.^96^ The decrease in the N 1s BE (by ∼0.2–0.4 eV) for higher electron doses is also correlated with the C 1s signal behavior, as is expected assuming that destruction of the dipole layer via the C–Au bond termination causes the change in the electrostatic energy, affecting both the C and N atoms of the monolayer. Thus, the similar character of the N 1s and C 1s data as a function of irradiation dose additionally supports the proposed model of electron-beam-induced NHC SAMs structure modification. Moreover, fitting the N 1s data presented in Figure 3c and Figure S4 at the dose range where the cross-linking process is saturated (above ∼10 mC/cm^2^) provides another, and independent, estimation for the respective cross section for the C–Au bond termination (σ_Au-C_) which for all NHC monolayers analyzed here is in the same range as calculated above from the respective C 1s data, *i.e*., ∼10^–18^ cm^2^ (see Table 1).

**Figure 3:**
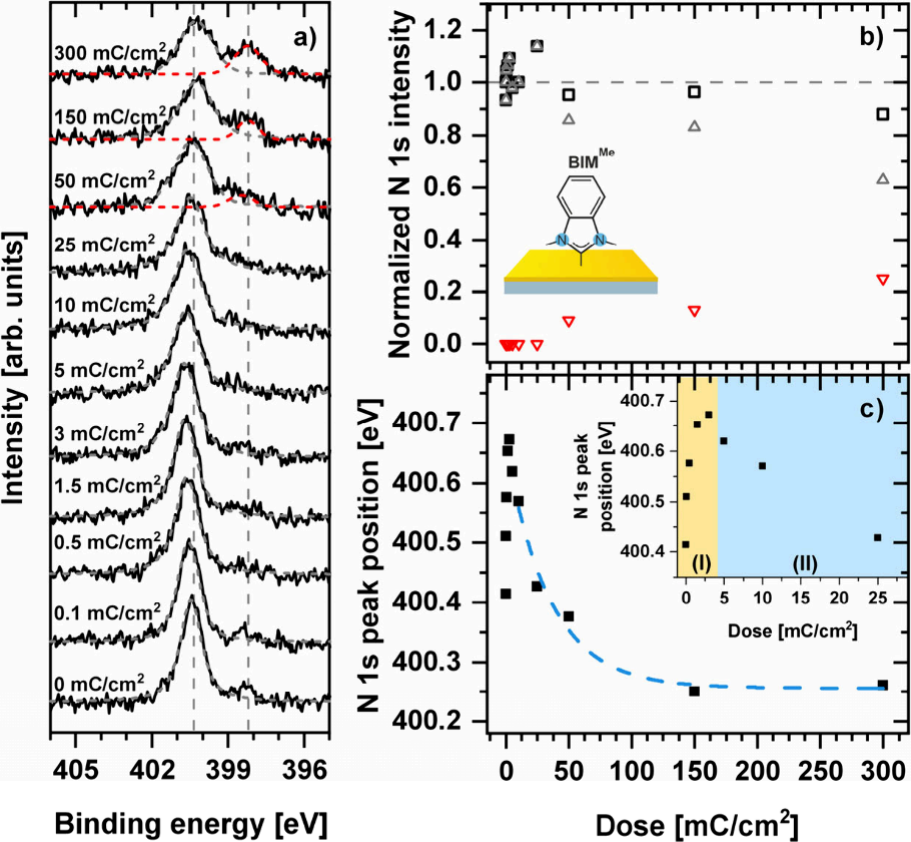
(a) N 1s XPS data for electron-beam (50 eV) irradiated BIM^Me^/Au monolayers using electron doses in the 0–300 mC/cm^2^ range. The gray dashed lines indicate peak position (∼400.4 eV) for nonirradiated (native) sample and position of the new peak (∼398.3 eV) visible only at higher irradiation doses. (b) Normalized intensity of total N 1s signal (black square), main component at ∼400.5 eV (gray triangles), and new component at ∼399–398 eV (red triangles) for BIM^Me^/Au monlayer as a function of the electron dose. (c) BE of the main component of N 1s as a function of the electron dose for BIM^Me^/Au. The inset shows close-up for low doses indicating increase (by the cross-linking process–yellow range) and decrease (by the C–Au bond termination, blue range) of the BE value. The blue dashed line in panel c shows fitting of the saturation function (an analogue of eq 1) to the data in the 10–300 mC/cm^2^ range where the C–Au bond termination process takes place.

The overall process of electron-beam-induced NHC SAMs modification is presented schematically in the upper panel of Figure 4, including two stages of this process, i.e. (i) the cross-linking and (ii) C–Au bond termination and N–Au bond formation. To test the possible application of NHC SAMs for carbon nanomembrane (CNMs) formation, the monolayers irradiated with the total dose of 150 mC/cm^2^ was delaminated from the Au substrates using PMMA-based protocol^46,97^ and transferred onto holey TEM grids, as schematically indicated in the upper panel of Figure 4. The quality of the resulting freestanding CNMs obtained from all types of NHC SAMs analyzed here was monitored by scanning electron microscopy (SEM). Our SEM data presented in Figure 4 show the formation of continuous CNMs in the case of BIM^Me^/Au and NIM^Me^/Au SAMs. In contrast, for the other two systems analyzed here, i.e., IM^Me^/Au and BIM^iPr^/Au SAMs, the continued CNMs are not formed, revealing pores with dimensions of ∼50–250 nm. These pores are a result of the low values of the cross sections discussed earlier and presented in Table 1. For the IM^Me^/Au, the formation of nanopores is caused also by too short molecules forming this type of monolayer and thus a lower mechanical stability of the resulting CNM, which then can be easily ruptured during the delamination process and subsequent transfer onto the TEM grid. For BIM^iPr^/Au, the length of the molecules forming this monolayer is expected to be sufficient, as confirmed by the formation of continuous CNMs obtained for analogues BIM^Me^/Au monolayer. However, a continued CNM is not formed, resulting from the increased size of the intermolecular distance between the molecules and thus reduced packing density due to the presence of the side groups which limit the efficiency of the cross-linking process, as discussed earlier. Generally, the low quality of the IM^Me^ and BIM^iPr^ CNMs is most likely related to the low lateral density of carbon atoms in cyclic structures which can participate in the cross-linking; that is, for IM^Me^ (13.0 C/nm^2^) and BIM^iPr^ (15.3 C/nm^2^), this parameter is significantly lower with respect to BIM^Me^ (23.9 C/nm^2^) and NIM-Me (40.0 C/nm^2^). In future work, more NHC SAMs with varying packing density will be tested; however, the current study already shows that irradiation of low packing density NHC structures leads to the mechanically unstable CNM fabrication.

**Figure 4:**
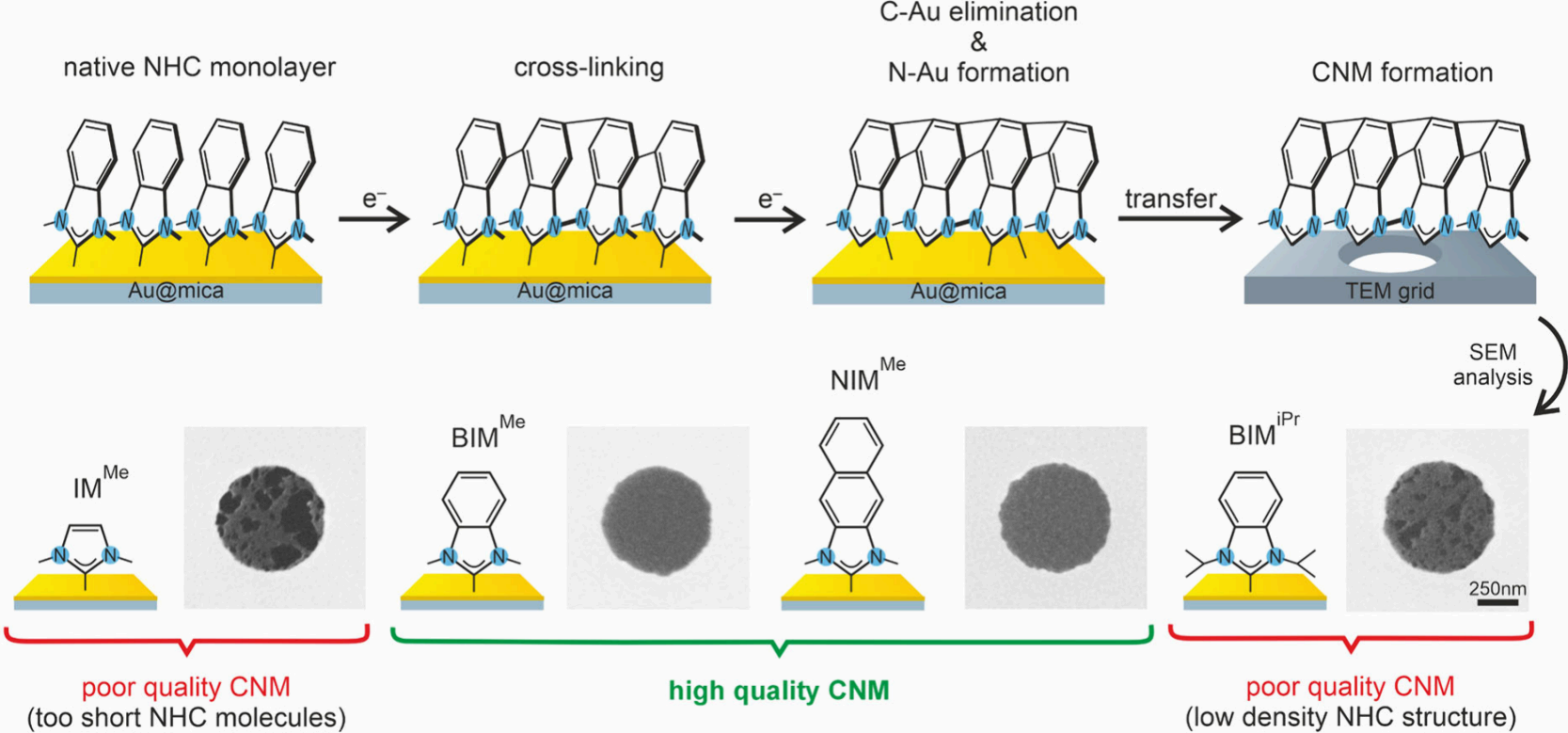
Upper panels show schematic process of electron beam induced NHC SAMs modification and subsequent fabrication of the carbon nanomembranes (CNMs) that includes the following steps: (i) irradiation induced cross-linking, (ii) C–Au bonds elimination and N–Au bond formation, and (iii) delamination of the monolayer and transfer on the TEM grid. The lower panels show representative SEM images of freestanding CNMs transferred onto holey TEM grid and obtained by irradiation of respective NHC SAM using 50 eV electron beam and total irradiation dose of 150 mC/cm^2^. Continuous CNM are formed from BIM^Me^/Au and NIM^Me^/Au SAMs. Mesh-like CNMs with high density of large nanopores (dark patches in SEM images) are obtained for either short IM^Me^/Au or low-density BIM^iPr^/Au monolayers.

In summary, detailed XPS analysis of electron-irradiated NHC SAMs (IM^Me^/Au, BIM^Me^/Au, NIM^Me^/Au, and BIM^iPr^/Au) revealed the basic mechanism of modification of these systems by a low-energy (50 eV) beam and its dependence on the precursor structure. Our analysis shows that similar to other aromatic SAMs, the NHC monolayers undergo a cross-linking process with a relatively small reduction of the film thickness (∼20–10%), even for the systems which are exclusively based on using an imidazolium unit without any aromatic moiety. The measured cross section for the film thickness reduction (∼10^–16^ cm^2^), which is higher for systems with higher efficiency of the cross-linking process, grows with the number *n* (*n* = 0–2) of phenyl rings in the molecules forming NHC monolayers (IM^Me^/Au < BIM^Me^/Au < NIM^Me^/Au). At the same time, this parameter, and thus the cross-linking process, is strongly reduced for analogues NHC monolayers (BIM^iPr^/Au < BIM^Me^/Au) where short methyl (Me) side groups were substituted by a bulkier isopropyl (iPr). In both cases, such behavior can be rationalized assuming a higher cross-linking probability for longer aromatic molecules and lower cross-linking probability for larger separation between molecules controlled by the size of the side groups, respectively. Another process, which takes at higher doses of electron irradiation, is elimination of the C–Au bonds between the molecules, forming NHC SAM and the Au substrate. Importantly, the measured cross section for this process (∼10^–18^ cm^2^) is lower by 1 or 2 orders of magnitude compared to aromatic SAMs based on thiol or carboxylic anchoring group, indicating much higher stability toward electron irradiation of the C–Au bond compared to S–Au or O–Au, respectively. The estimated fraction of the C–Au anchoring groups, which are eliminated in the NHC SAMs, also depends on their structure at the molecule–metal interface and is higher for IM^Me^/Au and BIM^iPr^/Au compared to BIM^Me^/Au and NIM^Me^/Au following also the former analysis of their thermal stability. Together with elimination of the original C–Au bond, our data indicate that during irradiation, a new bonding with the substrate is activated via formation of the N–Au bond, which is a result of electron-induced modification of the imidazolium moiety. Finally, the analysis of delamination and transferring of electron-irradiated NHC monolayers on a holey TEM grid reveals formation of freestanding CNMs. Continuous CNMs are formed for the BIM^Me^/Au and NIM^Me^/Au systems. The application of shorter molecules (IM^Me^/Au) or having more bulky nitrogen side groups (BIM^iPr^/Au) results in a high density of nanopores and the fabrication of mesh-like CNMs. These observations are fully correlated with the structurally controlled efficiency of the cross-linking process, which determines the mechanical stability of the CNMs during their delamination and transferring.

To conclude, our results provide design rules to optimize the NHC SAMs structure for their effective modification by electron irradiation. Such optimization becomes particularly interesting considering that NHC monolayers provide much higher stability of their bonding with the metal substrate toward electron irradiation compared to standard thiols or recently investigated carboxylic acids. Thus, NHC monolayers offer an interesting alternative for chemical lithography where structural modification of SAMs by electron or photon beams (considering that the primary damage in SAMs induced by X-ray, UV, or EUV irradiation is due to photo and secondary electrons)^23,98−100^ should be limited mainly to the terminal group, keeping bonding with the substrate mostly intact.^29,36,38,101,102^ Another important advantage of NHC SAMs in the context of electron-induced modification, which is also highly sensitive to their design and packing density, is their application for sulfur-free CNM fabrication as a much more stable alternative for recently proposed carboxylic SAMs.^46−49^

## Supporting Information Available

Sample preparation, electron irradiation process, XPS analysis, and Figures S1–S4 as indicated in the text (PDF)
Transparent Peer Review report available (PDF)
