# Supplementary material for: Electron-Beam-Induced Modification of N-Heterocyclic Carbenes: Carbon Nanomembrane Formation
Source: J Phys Chem Lett. 2024 Aug 2;15(32):8196–204. doi: 10.1021/acs.jpclett.4c01705 (PMC11331524; doi:10.1021/acs.jpclett.4c01705)
Supplement: Supplementary file 1 — jz4c01705_si_001.pdf [file jz4c01705_si_001.pdf]

**Supporting Information for:**

# Electron Beam Induced Modification of N-Heterocyclic Carbenes – Carbon Nanomembrane Formation

*Daria M. Cegiełka,[1,2] Martha Frey,[3] Krzysztof Kozieł,[4] Christof Neumann,[3] Andrey Turchanin[3,5]* and Piotr Cyganik[1]**

[1]Jagiellonian University, Faculty of Physics, Astronomy and Applied Computer Science, Smoluchowski Institute of Physics, Łojasiewicza 11, 30-348 Krakow, Poland.

[2]Jagiellonian University, Doctoral School of Exact and Natural Sciences, Łojasiewicza 11, 30-348 Krakow, Poland

[3]Institute of Physical Chemistry, Friedrich Schiller University Jena, Lessingstraße 10, 07743 Jena, Germany

[4]Faculty of Chemistry, Jagiellonian University, 30-387 Krakow, Poland

[5]Jena Center for Soft Matter, 07743 Jena, Germany

*corresponding authors: piotr.cyganik@uj.edu.pl, andrey.turchanin@uni-jena.de

KEYWORDS: N-heterocyclic carbenes, self-assembled monolayers, electron irradiation, carbon nanomembrane.

**SAM Preparation.** The synthetic protocols for all NHC compounds (*i.e.* IM, $BIM^{Me}$, $BIM^{iPr}$ and NIM) used in this study (schematically presented in **Figure 1)** can be found elsewhere.[1] All NHC SAMs on Au(111) have been prepared strictly following former spectroscopic characterization of these systems.[1] In short, the Au(111) substates were prepared at 300 $^{o}$C by evaporation of ~100 nm (rate ~0.15 nm/s) of gold on freshly cleaved mica (grade V1, Ted Pella USA). Prior to the evaporation, the mica substrate was annealed at evaporation temperature for ~15 h to remove contaminations. Respective SAMs were prepared by immersion (~20 h) of Au(111) substrates in the solution (1 mM) of the respective compound in THF (anhydrous, Sigma-Aldrich) which was degassed prior to the incubation (5-6 cycles of freeze-pump-thaw procedure in the Schlenk apparatus). The incubation of samples was conducted at RT under controlled argon atmosphere in the glovebox (MBraun). Directly before analysis samples were taken out from solution, rinsed (pure THF) and dried under nitrogen stream.

**Electron Irradiation.** The irradiation experiments were performed using 50 eV electron beam generated by the NEK-SC 150 (Staib) electron gun in the same ultra-high vacuum system as used for the XPS analysis.

**X-ray Photoelectron Spectroscopy (XPS).** The analysis was conducted *in situ* using ultra-high vacuum (UHV, base pressure 2 x $10^{-10}$ mbar) system (Scienta Omicron) equipped with monochromatic X-ray source (Al $K_{\alpha}$) and electron energy analyzer (Argus CU) working at 0.6 eV spectral energy resolution. For the calibration of binding energy (BE) scale, the Au $4f_{7/2}$ peak (BE

= 84.0 eV) was used. For fitting obtained XP spectra Voigt functions were used after background subtraction using linear (N 1s) or Shirley (C 1s, Au 4f) function.

**The CNM formation.** The transfer of CNMs on TEM grids (Quantifoil R 2/2 on Cu, 400 mesh) was performed following protocol described previously.[2] In short the process consist of several consecutive steps that include: (1) spin-casting of poly(methyl methacrylate) (PMMA) on CNM/Au sample and baking on a hotplate at 90 °C for 5 min, (2) spin-casting second PMMA layer followed by baking with the same parameters, (3) electrochemical delamination (in NaOH, voltage of 2−3 V) of CNM from the Au substrate, (4) transferring the CNM/PMMA sandwich on TEM grid after cleaning the sample in ultrapure water, (5) drying at 50 °C for 1 h on a hotplate, (6) immersion in acetone followed by rinsing with isopropanol to remove the PMMA layer and (7) critical point drying (Autosamdri-815, Tousimis).

**Scanning Electron Microscopy (SEM).** The Zeiss Sigma VP field emission scanning electron microscope was used for collecting SEM images using the in-lens detector and an electron beam energy of 15 kV.

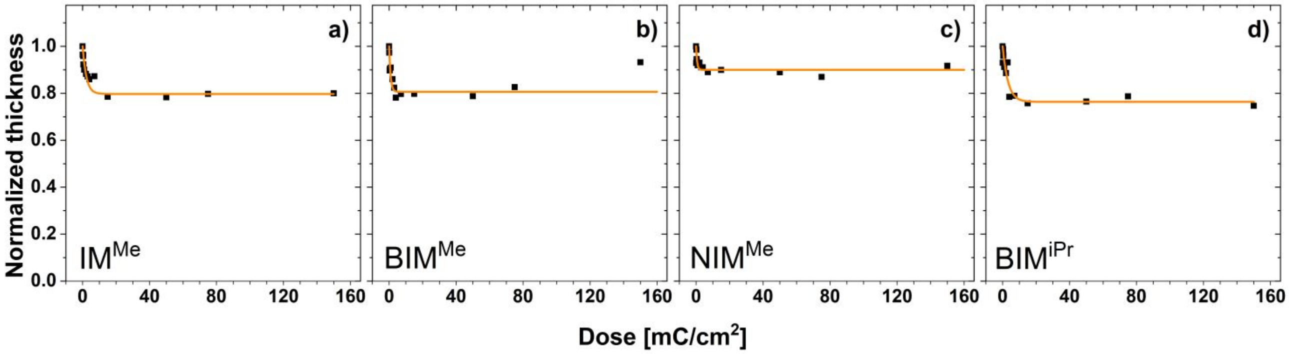


**Figure S1.** Normalized film thickness of NHC/Au monlayers as a function of the total electron dose with indicated (orange line) fitting of the saturation function.

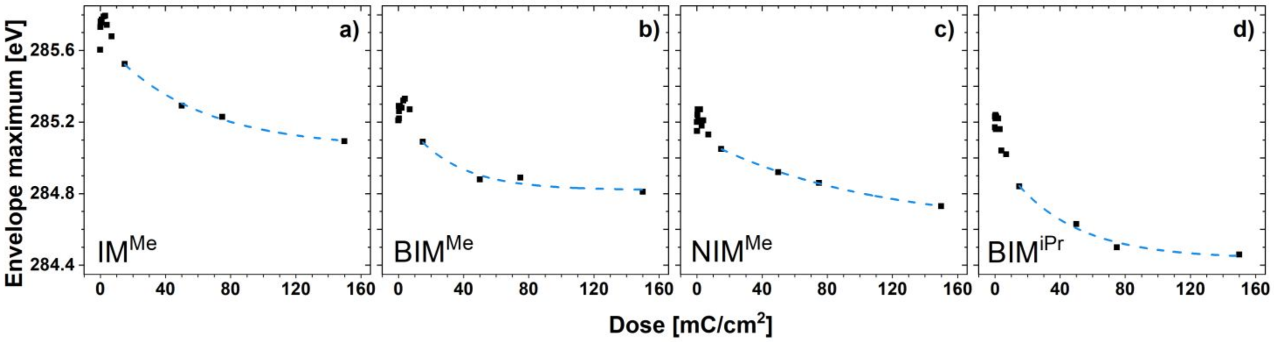


**Figure S2**. Binding energy (BE) of C 1s peak (envelope) maximum as a function of the total electron dose for NHC/Au monolayers. The blue dashed line shows fitting of the saturation function to the data in the range 15-150 mC/cm$^2$ where the Au-C bond termination process takes place (see the main text for details).

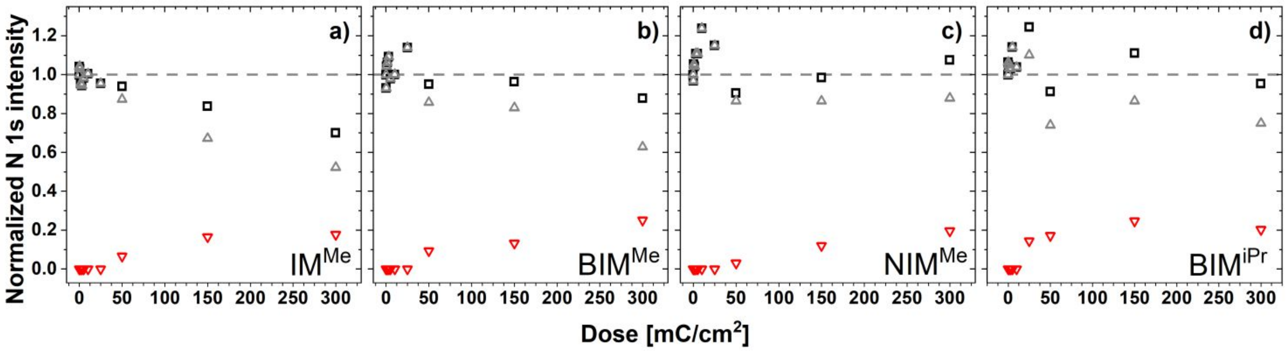


**Figure S3**. Normalized intensity of total N 1s signal (black square), main component at ~400.5 eV (grey triangles) and new component at ~399-398 eV (red triangles) for NHC/Au monlayer as a function of the total electron dose.

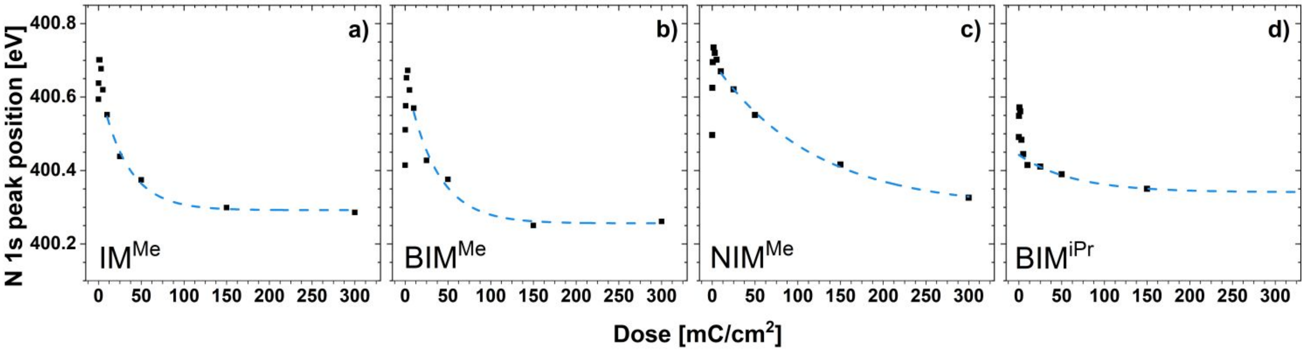


**Figure S4**. Binding energy (BE) of the main component of N 1s peak as a function of the total electron dose for NHC/Au monolayers. The blue dashed line shows fitting of the saturation function to the data in the range 10-300 mC/cm$^2$ where the Au-C bond termination process takes place (see the main text for details).

(1) Wróbel, M.; Cegiełka, D. M.; Asyuda, A.; Kozieł, K.; Zharnikov, M.; Cyganik, P. N-Heterocyclic Carbenes – The Design Concept for Densely Packed and Thermally Ultra-Stable Aromatic Self-Assembled Monolayers. *Nano Today* **2023**, *53*, 102024. https://doi.org/10.1016/j.nantod.2023.102024.

(2) Neumann, C.; Szwed, M.; Frey, M.; Tang, Z.; Kozieł, K.; Cyganik, P.; Turchanin, A. Preparation of Carbon Nanomembranes without Chemically Active Groups. *ACS Appl. Mater. Interfaces* **2019**, *11* (34), 31176–31181. https://doi.org/10.1021/acsami.9b09603.
