# Supplementary material for: Electron-Beam-Induced Modification of N-Heterocyclic Carbenes: Carbon Nanomembrane Formation
Source: J Phys Chem Lett. 2024 Aug 2;15(32):8196–204. doi: 10.1021/acs.jpclett.4c01705 (PMC11331524; doi:10.1021/acs.jpclett.4c01705)
Supplement: Supplementary file 2 — jz4c01705_si_002.pdf [file jz4c01705_si_002.pdf]

jz-2024-01705y.R1

Name: Peer Review Information for "Electron Beam Induced Modification of N-Heterocyclic Carbenes – Carbon Nanomembrane Formation"

First Round of Reviewer Comments

Reviewer: 1

Comments to the Author

The manuscript by D. Cegielka et al. is devoted to the modification of N-heterocyclic carbenes (NHCs) by electron irradiation and related implications. NHC monolayers have become recently very popular in view of their exceptional thermal and chemical stability, so that the subject of the manuscript is in the mainstream of the current research. The results are new, systematic, and well presented. The referencing is appropriate.

I suggest the acceptance of the manuscript after minor revisions (see below).

Specific comments:

General: The authors can consider to shorten the manuscript to some extent.

General: The authors should check the manuscript for grammatical errors.

General: many “the” and “,” are missing

Page 3: “some ultrafiltration process” – ”some ultrafiltration processes” or ”a specific ultrafiltration process”

Page 3: use either “anchoring group” (preferable) or “bonding group” over the manuscript

Page 4: “seems also crucial“ - “seems also to be crucial“

Page 5: “upright oriented, monolayers“ - “upright oriented monolayers“

Page 5: “formed by THE molecules”

Figure 2b: The details of the inset are not perceptible. It is enough to have the cross-section values in the table.

Page 9: “longest structure” – ”longest molecular backbone”

Page 9: The authors consider sigma-TH as a measure of the efficiency of crosslinking. This is probably true in view of the curve in Figure 2b. The sigma-TH shows how fast the film is sealed because of the crosslinking, so that the thickness cannot change any more. These considerations should be mentioned.

Page 12: “conformation of chemical bonds“ – “exact configuration of the chemical bonds”

Page 13: “photo electrons“ – ”photoelectrons”

Page 15: The low quality of the IM-Me CNMs is most likely related to the low density of the material (carbon atoms/nm^2 compared to graphene); see, e.g., the discussion in ref 33. Probably, the same is true for BIM-iPr, apart from the inefficient crosslinking. An estimate would be useful.

Figure 4: A contrast enhancement in the CNM images would be useful to emphasize the difference in the CNM quality.

Reviewer: 2

Comments to the Author

This paper describes e-beam lithography with NHC-based SAMs. The work needs a major revision to meet the high standard of the journal.

The mechanism on interaction of SAM with high-energy electron (e-beam) should be described more clearly with details. The current draft lacks the explanations although it is the key reaction.

Detailed explanations on CNM formation and transfer (shown in Supporting Information) must appear in the main texts as these are key steps for patterning and it imaging.

After the transfer of CNM film, the Au surface needs to be analyzed to make sure how much residues still remain on the surface. Again, this may be related to the quality of CNM such as surface roughness and edge roughness.

The following studies are patterning with SAM and directly related to this work. Therefore, this work must be cited: https://doi.org/10.1039/D3LF00248A; Chem. Mater. 2024, 36, 11, 5500–5507.

The authors claim the work is of interest to photolithography and nano-patterning, which is important for modern semiconductor industry. Related discussion must be added in the introduction part of this work with citing the following important papers: Polymer, 2023, 280, 126020; Coordination Chemistry Reviews, 2023, 493, 215307; https://doi.org/10.1021/acsmaterialsau.4c00010; Sci. Adv. 2023, 9, 3adf5997.

Figure S3 illustrating N1s XP signal variations as a function of dose does not show clear trends.

In the CNM formation step, the film was rinsed with water, not organic developer. I cannot fully understand this.

It has been reported that NHC SAM density varies depending on the methods to deposit molecules: J. Mater. Chem. A, 2023,11, 16233-16242; Nature Communications volume 11, Article number: 5714 (2020); Chem. Commun., 2022,58, 13188-13197. While these references need to be cited for discussing the density of NHC SAM, I suggest a control experiment varying the density to confirm density-patterning relationship (in this work or for future work).

Author's Response to Peer Review Comments:

Kraków 06.07.2024

Dear Professor Editor,

We would like to thank you, and both Reviewers, for a very positive reception of our manuscript. In the following part of this letter we provide our point-by-point replays to Reviewers comments, and questions, according to the requested revision of our paper. We hope that revised manuscript will be accepted for publication in The Journal of Physical Chemistry Letters.

Considering formal issues we note that:

1) The style of all references has been reformatted to JPC Letters
2) The pages in SI file have been numbered correctly
3) The TOC graphic was reformatted and placed properly in the manuscript

Sincerely yours,

Piotr Cyganik

Reviewer 1

The manuscript by D. Cegielka et al. is devoted to the modification of N-heterocyclic carbenes (NHCs) by electron irradiation and related implications. NHC monolayers have become recently very popular in view of their exceptional thermal and chemical stability, so that the subject of the manuscript is in the mainstream of the current research. The results are new, systematic, and well presented. The referencing is appropriate. I suggest the acceptance of the manuscript after minor revisions (see below).

Authors-reply

We thank the Reviewer for indicating the importance and the overall high quality of our paper.

Reviewer 1

General: The authors can consider to shorten the manuscript to some extent.
General: The authors should check the manuscript for grammatical errors.
General: many "the" and "," are missing

Authors-reply

The article length is at the upper limit of the JPCL format. Considering the comments of the second Reviewer who asks for even more elaborative description of irradiation processes we think that the length of the paper is well balanced in the current form. We went through the paper once again and made some small grammatic corrections indicated in the text.

Reviewer 1

Page 3: "some ultrafiltration process" – "some ultrafiltration processes" or "a specific ultrafiltration process"

Authors-reply

Corrected to *"some ultrafiltration processes"*

Reviewer 1

Page 3: use either "anchoring group" (preferable) or "bonding group" over the manuscript

Authors-reply

Corrected – along the whole paper we use now "anchoring group"

Reviewer 1

Page 4: "seems also crucial" - "seems also to be crucial"

Page 5: "upright oriented, monolayers" - "upright oriented monolayers"

Page 5: "formed by THE molecules"

Page 9: "longest structure" – "longest molecular backbone"

Page 12: "conformation of chemical bonds" – "exact configuration of the chemical bonds"

Page 13: "photo electrons" – "photoelectrons"

Authors-reply

Corrected

Reviewer 1

Figure 2b: The details of the inset are not perceptible. It is enough to have the cross-section values in the table.

Authors-reply

We prefer to have graphical presentation of data in **Figure 2b** as well. We would like to note that **Figure 2b** is two columns wide and the inset should be well-visible even in the printed paper.

Reviewer 1

Page 9: The authors consider sigma-TH as a measure of the efficiency of crosslinking. This is probably true in view of the curve in Figure 2b. The sigma-TH shows how fast the film is sealed because of the crosslinking, so that the thickness cannot change any more. These considerations should be mentioned.

Authors-reply

We fully agree with Reviewer comment. To make this statement more direct we have modified respective paragraph where sigma-TH is analyzed in the following way:

Was: *The higher the value of Df_{TH}, the lower is the dose of electron irradiation needed to reach the saturation range of film thickness reduction associated with the completeness of the monolayer cross-linking process.*

Now: *The higher the value of Df_{TH}, the lower is the dose of electron irradiation needed to reach the saturation range of film thickness reduction associated with the completeness of the monolayer cross-linking process. Therefore, the Df_{TH} can be considered as a measure of the cross-linking efficiency.*

Reviewer 1

Page 15: The low quality of the IM-Me CNMs is most likely related to the low density of the material (carbon atoms/nm^2 compared to graphene); see, e.g., the discussion in ref 33. Probably, the same is true for BIM-iPr, apart from the inefficient crosslinking. An estimate would be useful.

Authors-reply

As we underline in the paper (page 5 and 9), the IM-Me, BIM-Me and NIM-Me SAMs have similar high packing density (ref. 57). The fact that IM-Me SAMs does

not show high quality CNMs unlike BIM-Me and NIM-Me we attribute in the paper directly to lower thickness of this monolayer, and thus reduced chances for efficient cross-linking by transition of primary and secondary electrons for reaching sufficient level of mechanical stability during delamination and transferring of CNMs (page 9 and 15). In this case such difference could be correlated with density of carbon atom per nm^2 as pointed by the Reviewer and proposed in ref. 33 i.e. we get the much higher values for NIM-Me (47.3 C/nm2) and BIM-Me (30.7 C/nm2) as compared to IM-Me (21.5 C/nm2). However, this parameter becomes problematic when we try to include in such analysis the BIM-iPr since the obtained value (28.5 C/nm2) is similar as for BIM-Me (30.7 C/nm2) and could thus indicate high quality CNMs formation, which as we show, is not the case. This discrepancy is easy to understand considering that additional 4 carbon atoms in BIM-iPr, compared to BIM-Me, are from the aliphatic side groups which does not contribute efficiently to the cross-linking process. Therefore, this parameter cannot be used for the analysis of the complete series of our data.

Reviewer 1

Figure 4: A contrast enhancement in the CNM images would be useful to emphasize the difference in the CNM quality

Authors-reply

The contrast in CNMs images was increased.

Reviewer 2

The mechanism on interaction of SAM with high-energy electron (e-beam) should be described more clearly with details. The current draft lacks the explanations although it is the key reaction.

Authors-reply

The paper describes interaction of SAMs with low-energy electron beam (50 eV) not high-energy electron beam, and this mechanism is described on the basis of extended set of literature indicating all aspects of the observed film modifications. The paper length is at the limit of JPCL, and the opinion of the second Referee is that paper is even too detail. Therefore, we think, that our description is sufficient and placed in the paper together with the respective data analysis to optimize understanding of the text for the reader.

Reviewer 2

Detailed explanations on CNM formation and transfer (shown in Supporting Information) must appear in the main texts as these are key steps for patterning and it imaging.

Authors-reply

Although we agree that details of the CNM formation are important, the format of JPCL (letter) does not allow us for including experimental information into the

main text of the paper, and therefore, it was entirely moved to the Supporting Information.

Reviewer 2

After the transfer of CNM film, the Au surface needs to be analyzed to make sure how much residues still remain on the surface. Again, this may be related to the quality of CNM such as surface roughness and edge roughness.

Authors-reply

We thank the reviewer for pointing out this important issue and refer to our previous study (now cited in the paper as ref. 97), where the synthesis and transfer of CNM films from different molecular precursors on Au substrates with variable roughness was analysed in detail (see Nanoscale 12 (2020) 8656–8663, https://doi.org/10.1039/D0NR01084G). In brief, there is a correlation between the topological features of the formed CNMs and the substrate roughness. In this sense, the Au/mica substrates, as used for carbenes in our present work, result in the CNMs with lowest variation of the topographic feature. Moreover, there are no microscopic CNM residuals left on the substrate after the transfer if the precursor SAMs are completely crosslinked. A spectroscopic analysis of the substrate after the transfer appears to be unfeasible due to the airborne contaminations resulting from the contact to ambient condition.

Reviewer 2

The following studies are patterning with SAM and directly related to this work. Therefore, this work must be cited: https://doi.org/10.1039/D3LF00248A; Chem. Mater. 2024, 36, 11, 5500–5507.

Authors-reply

Both references related to pattering with SAMs were added to the introduction section.

Reviewer 2

The authors claim the work is of interest to photolithography and nano-patterning, which is important for modern semiconductor industry. Related discussion must be added in the introduction part of this work with citing the following important papers: Polymer, 2023, 280, 126020; Coordination Chemistry Reviews, 2023, 493, 215307; https://doi.org/10.1021/acsmaterialsau.4c00010; Sci. Adv. 2023, 9, 3adf5997.

Authors-reply

All papers indicated by the Reviewer are hardly related to SAMs and their application for nanolithography. However, we do cite two of them now in the summary part in the general context of electron induced damage during EUV irradiation (page 18).

Reviewer 2

Figure S3 illustrating N1s XP signal variations as a function of dose does not show clear trends.

Authors-reply

The N 1s signal is related to only two N atoms per molecule so its signal/noise ratio is obviously much lower as compared to C 1s signal despite using state of the art XPS machine. The main point of data presented in **Figure 3b** and in **Figure S3** is showing the rise of a new component (red triangles) in the case of large electron irradiation dose (150 and 300 mC/cm2) related to N-Au bond formation, and this is clearly visible.

Reviewer 2

In the CNM formation step, the film was rinsed with water, not organic developer. I cannot fully understand this.

Authors-reply

Detachment of the CNM/PMMA sandwich from the Au substrate was realized by the electrochemical delamination procedure in a 0.2 M NaOH solution as described in Nanoscale 12 (2020) 8656–8663, https://doi.org/10.1039/D0NR01084G in detail (see also SI page S3). This reference is now included as ref. 97 into the revised manuscript. Rinsing with ultrapure water was used to remove possible NaOH residuals from the bottom CNM surface. Next, by preparing free-standing CNMs, the PMMA layer on the top CNM surface was dissolved in acetone and in superctitical CO_2 as described in the above-mentioned reference as well as in the SI page S3.

Reviewer 2

It has been reported that NHC SAM density varies depending on the methods to deposit molecules: kang; Nature Communications volume 11, Article number: 5714 (2020); Chem. Commun., 2022,58, 13188-13197. While these references need to be cited for discussing the density of NHC SAM, I suggest a control experiment varying the density to confirm density-patterning relationship (in this work or for future work).

Authors-reply

The issue of NHC SAMs packing density impact on CNMs formation is directly addressed in our paper by using model systems for which we have recently calculated packing density (molecule footprint) using dedicated approach in XPS (see Nano Today 2023, 53, 102024 – ref. 57). We show, and discuss, in the paper comparing BIM-Me and BIM-iPr that reduced (roughly by half) packing density, due to the increased side groups, handicaps formation of continuous, well-defined CNMs. Certainly in the future work more NHC SAMs with varying packing density will be tested for NHC formation, however, already current study clearly show that diluted structures lead to mechanically unstable CNMs formation. The additional references indicated by the Reviewer are now in the paper with the following modification of two short paragraphs:

Was (page 9): *As pointed out earlier, the modification of this side group from Me to iPr leads to almost twice lower packing density,*[59,72] *thus hindering the cross-linking process taking place between neighboring molecules in the monolayer.*

Now (page 9): *As pointed out earlier, the modification of this side group from Me to iPr leads to almost twice lower packing density,*[59,72] *thus hindering the cross-linking process taking place between neighboring molecules in the monolayer. This observation is particularly important considering that the packing density of NHC SAMs also depends on their preparation method i.e. in solution, in vacuum, or electrochemical.*[59,63,70,86]

Was (page 16): *However, a continues CNM is not formed, resulting from the increased size of the intermolecular distance between the molecules, due to the presence of the side groups which are limiting the efficiency of the cross-linking process, as discussed earlier.*

Now (page 16): *However, a continues CNM is not formed, resulting from the increased size of the intermolecular distance between the molecules, and thus reduced packing density, due to the presence of the side groups which are limiting the efficiency of the cross-linking process, as discussed earlier. In the future work, more NHC SAMs with varying packing density will be tested, however, the current study already shows that irradiation of low packing density NHC structures leads to the mechanically unstable CNMs fabrication.*

jz-2024-01705y.R2

Name: Peer Review Information for "Electron Beam Induced Modification of N-Heterocyclic Carbenes – Carbon Nanomembrane Formation"

Second Round of Reviewer Comments

Reviewer: 2

Comments to the Author

I appreciate the authors for thoroughly addressing the provided comments. The revised work is well-crafted and ready for publication. I enjoyed reading it. Congratulations on interesting results!

Reviewer: 1

Comments to the Author

I am mostly satisfied with the revision performed by the authors and their replies to my comments. I think that the paper can be accepted now.

I suggest, however, two further minor revisions, which I consider as optional but which can be useful to improve the quality of the manuscript. Further revision is in any case not necessary.

Figure 2: It looks as a 1.5 column figure to me. One can, however, make it a two-column one by arranging all three panels in a line, which will allow each panel to be printed larger, so that the insets will be better perceptible. The only thing to do is to reduce the number of spectra in Figure 2a (a reasonable thing to do in my opinion), to get the same height of this panel as (b) and (c).

My previous comment to page 15: I agree with the reply by the authors, which only underlines the correctness of my comment. But - these considerations should be introduced into the paper, which will not take much space but is quite important to understand the experimental data.

Something as: “The low quality of the IM-Me CNMs is most likely related to the low lateral density of material (21.5 C/nm2) with respect to BIM-Me (30.7 C/nm2) and NIM-Me (47.3 C/nm2). Interestingly, the density of BIM-iPr (28.5 C/nm2) is similar to that of BIM-Me, but 4 carbon atoms in BIM-iPr have the aliphatic character and, therefore, do not contribute efficiently to the cross-linking process.”

Author's Response to Peer Review Comments:

Kraków 13.07.2024

Dear Professor Editor,

We would like to thank you, and both Reviewers, for a very positive reception of our revised manuscript. In the following part of this letter we provide our point-by-point replays to Reviewers comments/suggestions. We hope that revised manuscript will be accepted for publication in The Journal of Physical Chemistry Letters.

Sincerely yours,

Piotr Cyganik

Reviewer 1

I appreciate the authors for thoroughly addressing the provided comments. The revised work is well-crafted and ready for publication. I enjoyed reading it. Congratulations on interesting results!

Authors-reply

We thank the Reviewer for such an enthusiastic reception of our work.

Reviewer 2

I am mostly satisfied with the revision performed by the authors and their replies to my comments. I think that the paper can be accepted now.
I suggest, however, two further minor revisions, which I consider as optional but which can be useful to improve the quality of the manuscript. Further revision is in any case not necessary.

Authors-reply

We would like to thank the Reviewer for his very positive reception of our revised paper. In the following we respond to his/her suggestions.

Reviewer 2

Figure 2: It looks as a 1.5 column figure to me. One can, however, make it a two-column one by arranging all three panels in a line, which will allow each panel to be printed larger, so that the insets will be better perceptible. The only thing to do is to reduce the number of spectra in Figure 2a (a reasonable thing to do in my opinion), to get the same height of this panel as (b) and (c).

Authors-reply

We would like to avoid not showing part of our data which would be necessary to follow suggestions of the Reviewer regarding modification of Figure 2. Moreover, this modification would be also necessary for Figure 3 which has the same structure as Figure 2 to allow for better data comparison. Therefore, we would like to keep both Figures in the present form.

Reviewer 2

My previous comment to page 15: I agree with the reply by the authors, which only underlines the correctness of my comment. But - these considerations should be introduced into the paper, which will not take much space but is quite important to understand the experimental data.
Something as: "The low quality of the IM-Me CNMs is most likely related to the low lateral density of material (21.5 C/nm2) with respect to BIM-Me (30.7 C/nm2) and NIM-Me (47.3 C/nm2). Interestingly, the density of BIM-iPr (28.5 C/nm2) is similar to that of BIM-Me, but 4 carbon atoms in BIM-iPr have the aliphatic character and, therefore, do not contribute efficiently to the cross-linking process."

Authors-reply

We have added the following section at the end of page 16.

"Generally, the low quality of the IM^{Me} and BIM^{iPr} CNMs is most likely related to the low lateral density of carbon atoms in cyclic structures which can participate in the cross-linking i.e. for IM^{Me} (13.0 C/nm^2) and BIM^{iPr} (15.3 C/nm^2) this parameter is significantly lower with respect to BIM^{Me} (23.9 C/nm^2) and NIM-Me (40.0 C/nm^2)."
